# Alterations in the brain adenosine metabolism cause behavioral and neurological impairment in ADA-deficient mice and patients

**DOI:** 10.1038/srep40136

**Published:** 2017-01-11

**Authors:** Aisha V. Sauer, Raisa Jofra Hernandez, Francesca Fumagalli, Veronica Bianchi, Pietro L. Poliani, Chiara Dallatomasina, Elisa Riboni, Letterio S. Politi, Antonella Tabucchi, Filippo Carlucci, Miriam Casiraghi, Nicola Carriglio, Manuela Cominelli, Carlo Alberto Forcellini, Federica Barzaghi, Francesca Ferrua, Fabio Minicucci, Stefania Medaglini, Letizia Leocani, Giancarlo la Marca, Lucia D. Notarangelo, Chiara Azzari, Giancarlo Comi, Cristina Baldoli, Sabrina Canale, Maria Sessa, Patrizia D’Adamo, Alessandro Aiuti

**Affiliations:** 1 San Raffaele Telethon Institute for Gene Therapy (SR-TIGET), IRCCS San Raffaele Scientific Institute, Milan, Italy; 2 Neurology Unit, Neurology Department, IRCCS San Raffaele Hospital, Milan, Italy; 3 Dulbecco Telethon Institute at Division of Neuroscience, IRCCS San Raffaele Scientific Institute, Milan, Italy; 4 Department of Pathology, University of Brescia, Brescia, Italy; 5 Psychological Service, Neurological Department, IRCCS San Raffaele Hospital, Milan, Italy; 6 Imaging Core and Neuroradiology Unit, Head and Neck Department, IRCCS San Raffaele Hospital, Milan, Italy; 7 Department of Medical Biotechnologies, University of Siena, Italy; 8 U.O.C. Clinical Pathology, AOUS, Siena, Italy; 9 Pediatric Immunohematology and Bone Marrow Transplantation Unit, IRCCS San Raffaele Hospital, Milan, Italy; 10 Vita-Salute San Raffaele University, Milan, Italy; 11 Neurophysiology Center, IRCCS San Raffaele Scientific Institute, Milan, Italy; 12 Department of Neurosciences, Psychology, Drug Research and Child Health, University of Florence, Florence, Italy; 13 Department of Molecular and Translational Medicine, Pathology Unit, University of Brescia, Brescia, Italy; 14 Neuroradiology Unit, IRCCS San Raffaele Scientific Institute, Milan, Italy; 15 Multimedica hospital, Neurological Rehabilitation, Limbiate, Italy

## Abstract

Adenosine Deaminase (ADA) deficiency is an autosomal recessive variant of severe combined immunodeficiency (SCID) caused by systemic accumulation of ADA substrates. Neurological and behavioral abnormalities observed in ADA-SCID patients surviving after stem cell transplantation or gene therapy represent an unresolved enigma in the field. We found significant neurological and cognitive alterations in untreated ADA-SCID patients as well as in two groups of patients after short- and long-term enzyme replacement therapy with PEG-ADA. These included motor dysfunction, EEG alterations, sensorineural hypoacusia, white matter and ventricular alterations in MRI as well as a low mental development index or IQ. Ada-deficient mice were significantly less active and showed anxiety-like behavior. Molecular and metabolic analyses showed that this phenotype coincides with metabolic alterations and aberrant adenosine receptor signaling. PEG-ADA treatment corrected metabolic adenosine-based alterations, but not cellular and signaling defects, indicating an intrinsic nature of the neurological and behavioral phenotype in ADA deficiency.

Mutations in the adenosine deaminase (ADA) gene are among the most common causes for severe combined immunodeficiency (SCID). ADA catalyzes the deamination of adenosine and deoxyadenosine. When absent, the systemic metabolic toxicity of these and other purine metabolites, is associated with SCID, organ damage and neurological alterations^1^,^2^. Without treatment, the condition is fatal and necessitates early intervention. Currently available treatments include allogeneic hematopoietic stem cell transplant (HSCT), enzyme replacement therapy (ERT) with bovine ADA (PEG-ADA) and hematopoietic stem cell gene therapy (HSC-GT)^3^.

Several non-immune abnormalities have been described in ADA-deficiency, including skeletal alterations^4^, lung alterations^5^,^6^, hepatic and renal disease^7^, indicating that it should be considered a ‘systemic’ metabolic disorder^8^,^9^. Moreover, neurological abnormalities^8^ and behavioral impairments^10^,^11^, reduced verbal expression, learning disability, hyperactivity, attention deficits, seizures and hearing deficits^8^,^12^ have been reported in patients surviving after bone marrow transplant or HSC-GT. Two reports showed that ADA-SCID patients after HSCT are at high risk of CNS complications^11^,^13^. Also ADA-SCID patients after HSC-GT continue to present with mild neurologic impairments^12^. However, previous studies were unable to identify transplantation-related or SCID-specific factors correlating with this neurologic outcome. Hence their pathogenesis or the underlying metabolic and molecular mechanisms remained unknown and insufficient data were available to assess whether treatment is efficient in preventing or controlling these alterations.

The effects of PEG-ADA on immune reconstitution and the metabolic alterations in ADA-SCID are well described, but its long-term effect on the neurological manifestations remained unclear. Children with ADA-deficiency proceed to HSCT or HSC-GT when appropriate, ERT is therefore discontinued and systemic long-term data are lacking. Moreover, behavioral studies are difficult to perform across different countries and continents^14^. Since complications from infections usually predominate their clinical presentation, it is extremely difficult to define whether the neurological impairments are primarily determined by the lack of ADA^10^,^15^.

It was hypothesized that the described neurological manifestations arise from an effect of adenosine and its derivatives on the nervous system^16^. Both adenosine and ATP have been implicated in mood and motivation behavior^17^. Moreover, there is rapidly growing literature about the involvement of purinergic signalling in most disorders of the CNS, such as neuropsychiatric and mood disorders^18^. Adenosine acts as a neuromodulator through a family of purinergic G-protein-coupled receptors^19^. Four different Adenosine receptors (Adora1, Adora2a, Adora2b and Adora3 receptor) have been identified^20^. The high affinity Adora1 and Adora2a are the most abundant in the nervous system and the most relevant under physiologic conditions^21^.

Given the complex nature and ubiquitous distribution of the adenosine (receptor) system, any imbalance can be expected to lead to neurological disease^16^. The neurological defects described in ADA-deficiency could be mediated by adenosine, deoxyadenosine or their derivates. In order to obtain new insights into the role of ADA in brain function and the impact of adenosine accumulation, we assessed neurological and behavioral features in ADA-SCID patients and Ada−/− mice. Moreover, we assessed the extent of correction after PEG-ADA treatment, which allowed the separation of metabolic from intrinsic cellular defects contributing to the ADA-SCID neurological and behavioral phenotype.

## Results

### Neurological abnormalities in ADA-SCID patients

We retrospectively analyzed 21 ADA-SCID patients (14 males, 7 females) referred to San Raffaele Hospital (Table 1). About half were born to consanguineous parents. Five were untreated at first evaluation (mean age: 1.2 years). 16 patients underwent variable periods of ERT with PEG-ADA. Treated patients were divided into two groups according to their age: 8 patients with less than 3 years of age (<3yrs, mean age: 1.5 years) and 8 patients aged more than 3 years (>3yrs, mean age: 13.1 years). Patients in the younger PEG-ADA-treated group initiated treatment on average at 0.7 and were treated for 1.3 years. Patients older than 3 years of age on average initiated treatment at 0.7 and were treated for 12.6 years. ADA-SCID patients underwent clinical neurological evaluation of disease status and instrumental exams including electroencephalography (EEG), Visual Evoked Potentials (VEP), Brainstem Auditory Evoked Responses (BAER) and brain Magnetic Resonance (MR).

**Figure 1:**
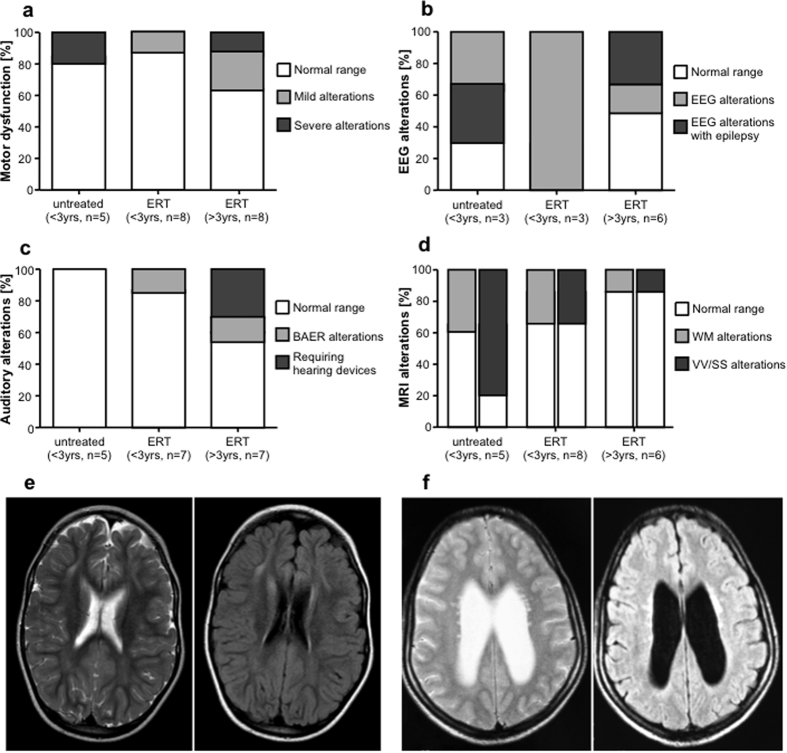
CNS dysfunctions in untreated ADA-SCID patients and under ERT. Neurological alterations in untreated patients (n = 5) and patients under ERT aged <3yrs (n = 8) and >3yrs (n = 7). (**a**) Percentage of patients with motor dysfunction such as coordination or deambulation deficits, alterations of muscle tone and trophism. Manifestation of one of these deficits is represented in light grey bars for mild alterations, whereas two or more deficits are shown as severe alterations in dark grey. (**b**) Percentage of patients with EEG alterations. Light grey bars represent alteration in EEG, whereas concomitant manifestation of epilepsy was considered as severe alterations (dark grey). (**c**) Percentage of patients with auditory alterations. Peripheral hearing loss detected by alterations in BAER potentials are represented in light grey bars, whereas clinically relevant deficits requiring hearing devices are shown in dark grey. (**d**) Light grey bars indicate percentage of patients with WM alterations in MRI. Dark grey bars indicate percentage of patients with abnormal size of ventricular system and subarchnoid spaces. (**e**) Representative MRI axial Spin Echo T2 (left panel) and FLAIR (right panel) images of patient 12. Diffuse, moderate enlargement of subarachnoid spaces. Diffuse signal alterations, hyperintense in T2 and FLAIR images, are seen in frontal corona radiata and posterior periventricular white matter (WM). (**f**) Representative MRI axial Spin Echo T2 (left panel) and FLAIR (right panel) images of patient 16. Enlargement of lateral ventricles. Multiple punctiform signal abnormalities, hyperintense on T2 and FLAIR images are evident in bilateral periventricular WM.

Severe motor dysfunctions were observed in one untreated ADA-SCID (20%) and one older PEG-ADA-treated patient (13%). Among the PEG-ADA-treated patients, one young (13%) and two older (25%) patients suffered from mild motor dysfunctions (Fig. 1a).

EEG resulted abnormal in 66% of untreated patients and in all three of the <3yrs-old PEG-ADA-treated patients group (100%), for which analyses were available. In the group of older PEG-ADA-treated patients, 50% showed an abnormal EEG. Two of them suffered from epilepsy and required anti-epileptic treatment (Fig. 1b). One untreated and one young PEG-ADA-treated ADA-SCID patient suffered from psychomotor retardation, mainly characterized by verbal delay.

All patients performed VEP that resulted normal except for two patients, which showed mild alterations (Table 1). One patient in the young and one in the older PEG-ADA-treated group showed mild BAER abnormalities, whereas two patients in the older PEG-ADA-treated group were affected by hearing loss requiring devices (Fig. 1c). These alterations alongside otolaryngological evaluation were diagnosed as sensorineural hypoacusia.

Brain MRI revealed leukoencephalopathy, enlargement of ventricles and subarachnoid spaces in all three groups of ADA-SCID patients (Fig. 1d, Supplementary Table 1). 40% of untreated patients showed white matter (WM) alterations in brain MR and 80% enlargement of ventricles and subarachnoid spaces (VV/SS). WM alterations were also detected in PEG-ADA-treated patients (38%/17%), whereas VV/SS enlargement was less common than in untreated patients (38%/17%). Representative MRI images of ADA-SCID patients with WM and VV/SS alterations are shown in Fig. 1e,f.

Overall, our data suggests that ADA-deficient patients commonly manifest several central nervous system defects. These form part of the disease phenotype and might be due to the systemic accumulation of adenosine. Despite partially reducing these manifestations, PEG-ADA treatment cannot completely prevent their onset or resolve pre-existing defects. The requirement for hearing devices appears only in the oldest patients, suggesting that some manifestations may worsen with age.

### Psychometric evaluation in ADA-SCID patients

**Figure 2:**
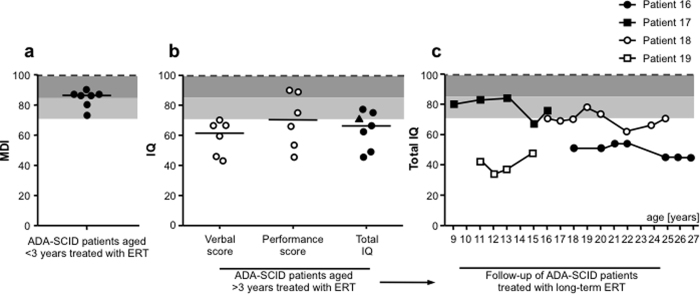
Neuropsychological alterations in ADA-SCID patients treated with ERT. PEG-ADA treated patients were divided into two groups of patients aged either less or more than 3 years of age. (**a**) Mental development index (MDI) as assessed by the Bayley Scale of Infant Development (2^nd^ Edition) in young ERT treated patients (n = 7); dotted line represents the population average, shaded areas represent −1SD/−2SD respectively. (**b**) Total intelligence quotient (IQ), derived from verbal and performance scores, as assessed in ERT treated patients aged more than 3 years (n = 7, mean ERT treatment: 12 years); dotted line represents the population average, shaded areas represent −1SD/−2SD respectively. One Patient, marked as triangle, was assessed as total IQ score only. (**c**) Development of total IQ score in 4 long-term ERT treated patients over time. Last shown timepoint for each patient corresponds to total IQ represented in panel b.

Seven patients of the younger and six of the older group of PEG-ADA-treated patients underwent neuropsychological assessment to measure their mental development index (MDI) or intelligence quotient (IQ) (Fig. 2). Equivalent analyses were unavailable for the group of untreated patients. PEG-ADA-treated patients aged <3yrs scored below but within 2 standard deviations (SD) of the population average MDI of 100 (Fig. 2a). Wechsler’s Intelligence Scales provided a total, verbal (VIQ) and performance (PIQ) intelligence quotient (IQ) for five patients, one patient was assessed as total IQ only (Fig. 2b). All patients’ VIQ resulted in the pathological range of below −2SD of the population average. The low VIQ fits with the verbal delay or deficit observed in most patients (Table 1). In contrast, the PIQ was higher in all patients, but nevertheless below the population average. The resulting total IQ in the older group of PEG-ADA-treated patients was well below the population average (<−1SD). In four long-term treated patients, their IQ was assessed over time (Fig. 2c). However no improvement occurred with long-term treatment and IQ scores remained <−1SD of the population average. All four patients are native Italians, thereby excluding a possible bias in VIQ due to use of a translator during assessment.

In addition to the quantitatively assessed psychometric scores, we observed a variety of unquantifiable behavioral aspects during longitudinal follow-up in ADA-SCID patients (see supplementary data).

### Morphological and histological analyses in the brain of Ada−/− mice

**Figure 3:**
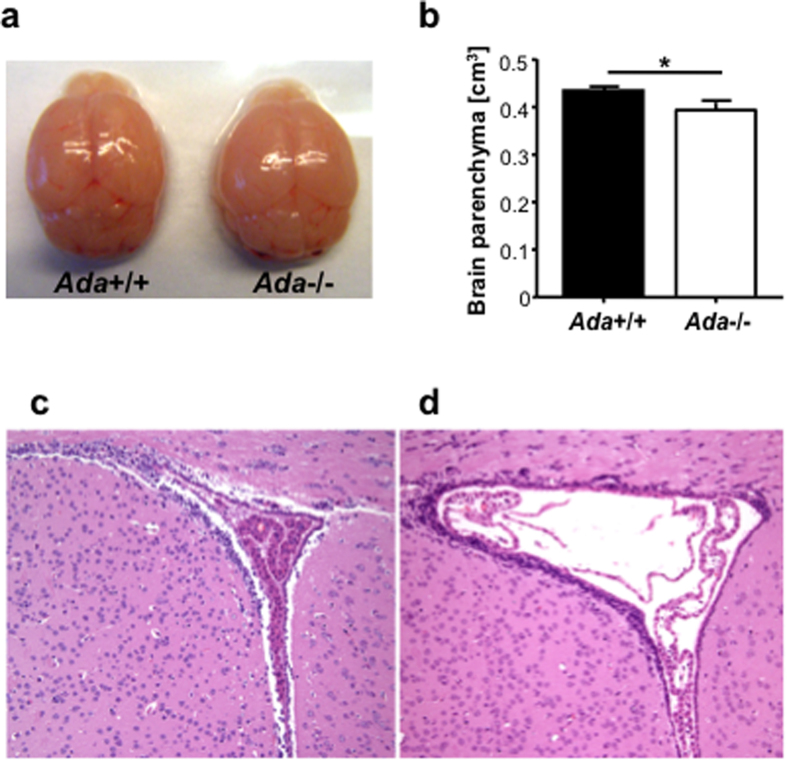
Alterations in the brain morphology of *Ada*−/− mice. (**a**) Brain explants from *Ada*−/− mice are smaller in size as compared to *Ada*+/+ brains. Upper view of *Ada*+/+ and *Ada*−/− brains at PND19. (**b**) Volume of brain parenchyma assessed by MRI of *Ada*+/+ (n = 6) and *Ada*−/− (n = 5) mice at PND20; *p < 0.05. Hematoxylin and Eosin staining of the lateral ventricles of 3 weeks old (**c**) *Ada*+/+ and (**d**) *Ada*−/−; 10x.

In order to dissect the cellular or metabolic mechanisms contributing to neurological and behavioral alterations in ADA-deficiency, we studied the Ada−/− mouse model that retains many features associated with ADA deficiency in humans, including systemic metabolic alterations and immunodeficiency^22^. Elevated adenosine levels cause abnormal alveolar development, leading Ada−/− mice to die post-natally within 3 weeks. Brains from Ada−/− mice are slightly reduced in size as compared to Ada+/+ (Fig. 3a). MRI imaging revealed a significant reduction of up to 10% brain parenchymal volume, but no additional alterations were observed in Ada−/− mice (Fig. 3b). Histological analyses on brains from 3-weeks-old Ada+/+ and Ada−/− mice (Fig. 3c,d), confirmed the previously described ventriculomegaly in Ada-deficient mice^23^, but showed but no other gross alterations. No alterations in myelination or neuronal loss were observed in ADA−/− mice by myelin (CNPase) and neuronal (NeuN) stainings (data not shown).

### Alterations in the brain purine metabolism in Ada−/− mice

ADA is ubiquitously expressed in all cell types, the highest level of ADA expression is found in lymphoid tissue, the gastrointestinal tract and the brain^24^,^25^.

**Figure 4:**
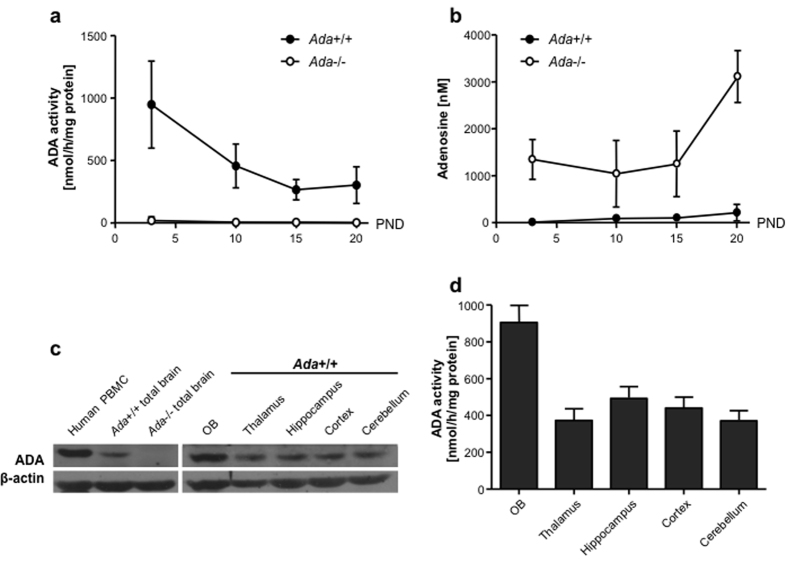
Alterations of the ADA metabolism in the brain of *Ada*−/− mice. (**a**) ADA activity/mg of total brain protein measured by HPCE-based analysis in total brain of *Ada*+/+ (n ≥ 8) and *Ada*−/− (n ≥ 10) mice at PND 3, 10, 15, 20. (**b**) Concentration of adenosine levels in total brain protein of *Ada*+/+ (n ≥ 8) and *Ada*−/− (n ≥ 10) mice at PND 3, 10, 15, 20. (**c**) Representative cropped Western Blot analysis for ADA (40 kDa) and β-actin (42 kDa) on brain from *Ada*+/+ and *Ada*−/− mice, positive control from human PBMCs. (**d**) ADA activity/mg of total protein in olfactory bulbs (n = 14), thalamus (n = 11), hippocampus (n = 13), cortex (n = 13) and cerebellum (n = 16) of *Ada*+/+ mice measured by HPCE-based analysis (average + SEM).

In order to assess Ada expression during early brain development, we measured Ada expression in wild-type mice from PND 3 until PND20. Ada expression in wild-type mice peaks at PND3 and decreases over time, whereas enzymatic activity is undetectable in Ada−/− mice (Fig. 4a). Since adenosine is a ubiquitous chemical messenger^26^, we measured accumulating adenosine levels in total brain of Ada−/− mice from PND3 until PND20. From birth until their death adenosine accumulated, while levels in Ada+/+ mice remained low (Fig. 4b). Presence and activity of ADA were also measured by Western Blot and ADA enzymatic assay (Fig. 4c,d) in different brain regions. Analyses show an even distribution in the thalamus, hippocampus, cortex and cerebellum, while the highest Ada expression was found in olfactory bulbs.

### Behavioral abnormalities in Ada−/− mice

Purinergic signaling in neurotransmission and neuromodulation is well established in the CNS, but few studies assessed its involvement in behavioral patterns^18^. To assess the ADA metabolism-specific contribution we tested Ada−/− and control littermates in a variety of developmental and behavioral tests. Littermates were subjected to the FOX battery to screen for sensory-motor development at PND 3, 6, 9, 12 and 18^27^. No significant differences were observed in Ada−/− mice for all tested parameters (Supplementary Table 2). At PND21, shortly before dying, Ada−/− mice showed small difficulties in some tests (crossed extensor reflex, negative geotaxis). Importantly, at the timepoint when most subsequent behavioral tests were carried out (PND15) Ada+/+ and Ada−/− performed equally.

Littermate mice were tested at PND15 and PND20 in the rotarod test (data not shown). No difference were observed for the time spent on the rotarod (PND15 ANOVA genotype effect: F[1,43] = 0.9, p = 0.35; PND20: F[1,42] = 2.94, p = 0.09), suggesting normal motor coordination. Only slight difficulties of individual Ada−/− mice were observed at PND20.

**Figure 5:**
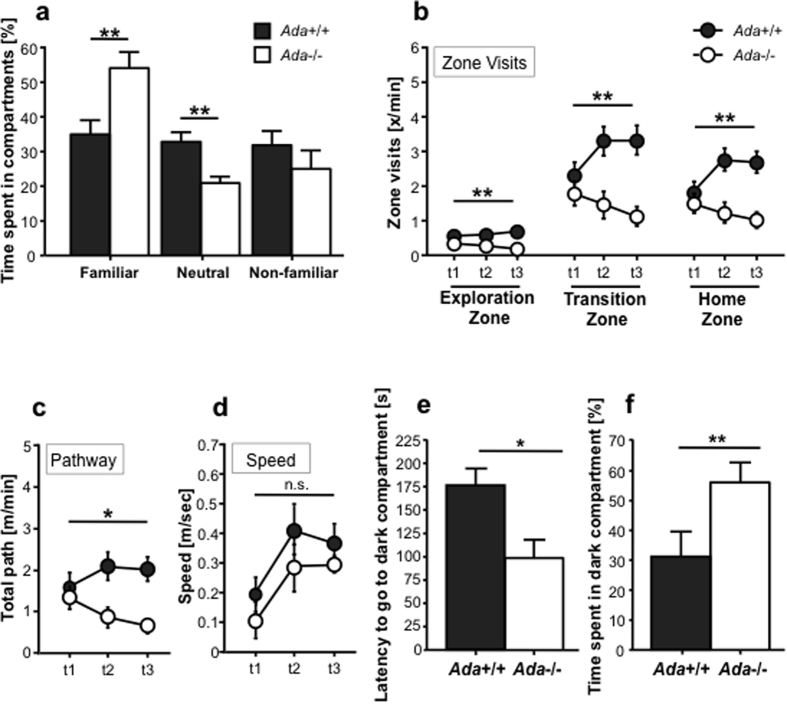
Behavioral differences between *Ada*+/+ and *Ada*−/− mice. (**a**) Olfactory discrimination task at PND20 between familiar, neutral and non-familiar compartments for *Ada*+/+ (n = 20) and *Ada*−/− (n = 24) mice as percentage of time spent in each compartment. Values represent the mean + SEM; **p < 0.005. (**b**) Zone visits during Open Field test of *Ada*+/+ (n = 25) and *Ada*−/− (n = 20) mice at PND15 (t1-3). (**c**) Total pathway and (**d**) speed during Open Field test of *Ada*+/+ (n = 25) and *Ada*−/− (n = 20) mice at PND15 (t1-3); n.s. = non significant. (**e**) Latency to go to the dark compartment during the dark and light test at PND15; ADA (n = 29) and *Ada*−/− (n = 33); *p < 0.05. (**f**) Time spent in the dark compartment during the dark and light test at PND 15; ADA (n = 29) and *Ada*−/− (n = 33); **p < 0.005.

To exclude olfactory impairment, which may influence behavioral performance, littermates were subjected to an olfactory discrimination task. Ada+/+ mice explored the three zones (familiar, neutral and non-familiar) similarly, whereas Ada−/− mice spent more time in the familiar zone (ANOVA genotype effect: F[1,42] = 10.42, p = 0.002; Fig. 5a), suggesting intact olfactory capability.

Two independent groups of littermates were tested at PND15 and PND20 in the open field test. PND15 Ada−/− mice showed significantly decreased activity, as assessed by the number of visits in the three defined zones of the arena (exploration, home and transition). We observed a significant reduction of zone visits in Ada−/− mice (exploration zone: PND15, genotype effect: F[1,43] = 9.25, p = 0.004; transition zone: PND15, genotype effect: F[1,43] = 9.34, p = 0.004 and PND20, genotype effect: F[1,23] = 8.59, p = 0.007; home zone: PND15, genotype effect: F[1,43] = 8.38, p = 0.006; Fig. 5b). The observed hypoactivity during the open field test correlates with a significantly reduced locomotor activity as shown by the total path covered (PND15, genotype effect: F[1,44] = 6.21, p = 0.016; Fig. 5c). The distance traveled was 52% reduced compared to control animals at PND15, but not due to a significant difference in velocity (Fig. 5d). These findings suggest a generalized hypoactivity in Ada−/− mice. Significantly reduced locomotor activity and hypoactivity were observed in Ada−/− mice also at PND20. However, it cannot be excluded that some respiratory distress further aggravated their hypoactivity (data not shown).

PND15 mice were also subjected to the dark and light test to monitor anxiety-like behavior. Their latency to go and the time spent in the dark compartment (safe zone) was significantly different. Ada−/− mice have a shorter latency to enter into the dark compartment (PND15, genotype effect: F[1,83] = 4.04, p = 0.02; Fig. 5e) and remain inside for a longer period of time (PND15, genotype effect: F[1,81] = 7.06, p = 0.01; Fig. 5f). These findings suggest that lack of Ada leads to alterations in explorative behavior in mice and anxiety-like behavior in an unfamiliar or aversive environment.

### Correction of metabolic but not behavioral defects in Ada−/− mice by ERT

We assessed whether the observed alterations and brain metabolism in Ada−/− mice could be corrected by systemic administration of ERT.

**Figure 6:**
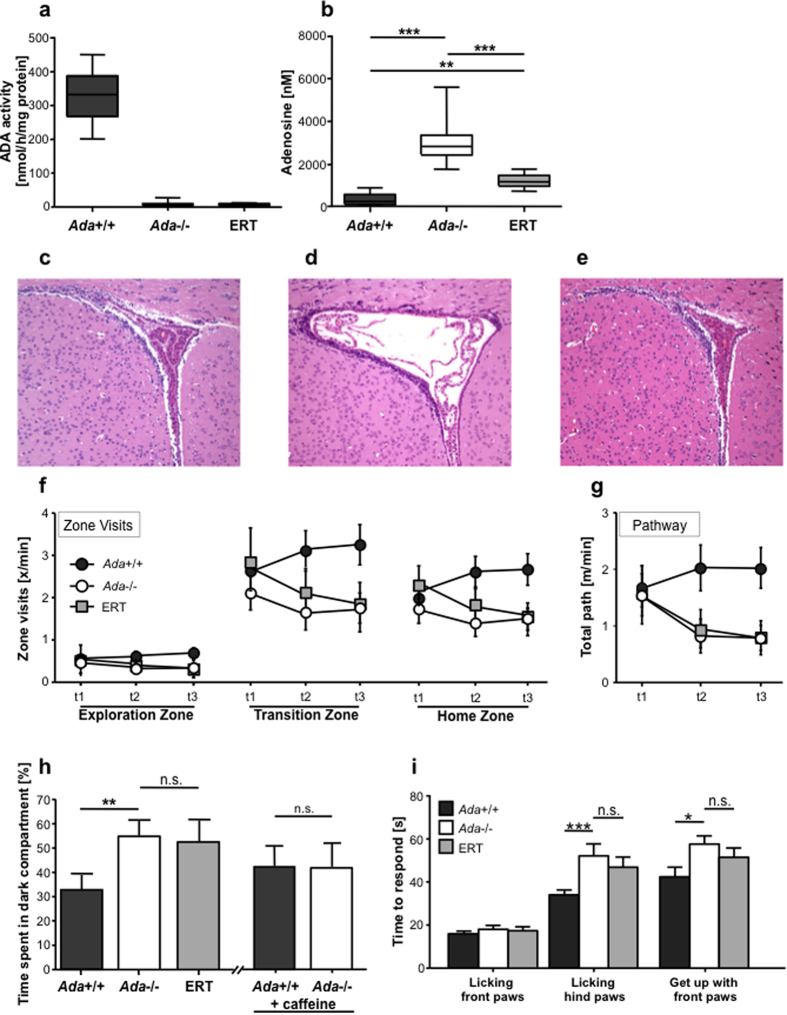
Correction of metabolic but not neurological alterations in ERT treated *Ada*−/− mice. (**a**) ADA activity/mg of total brain protein measured by HPCE-based analysis in 3 weeks old *Ada*+/+ (n = 9), *Ada*−/− (n = 11) and *Ada*−/− mice treated with ERT for two weeks (n = 10). (**b**) Concentration of adenosine levels in total brain from 3 weeks old *Ada*+/+ (n = 9), *Ada*−/− (n = 11) and *Ada*−/− mice treated with ERT for two weeks (n = 10); **p < 0.005; ***p < 0.0005. Hematoxylin and Eosin staining on the lateral ventricles of 3 weeks old (**c**) *Ada*+/+, (**d**) *Ada*−/− and (**e**) *Ada*−/− mice treated with ERT for two weeks; 10x. Panels Fig. 3c,d correspond to Fig. 2c,d. (**f**) Zone visits during Open Field test at PND15 (t1-3) of *Ada*+/+ (n = 25), *Ada*−/− (n = 20) and *Ada*−/− mice treated with ERT for two weeks (n = 8). (**g**) Total pathway during Open Field test at PND15 (t1-3) of 2 weeks old *Ada*+/+ (n = 25), *Ada*−/− (n = 20) and *Ada*−/− mice treated with ERT for one week (n = 8). (**h**) Time spent in the dark compartment during the Dark and Light Box test at PND15 of *Ada*+/+ (n = 29), *Ada*−/− (n = 33) and *Ada*−/− mice treated with ERT for one week (n = 8); **p < 0.005; time spent in the dark compartment in *Ada*+/+ (n = 20) and *Ada*−/− (n = 15) treated with daily i.p. injections with 50 mg/kg of caffeine; n.s. = non significant. (**i**) Hot plate latency to lick front and hind paws and to get up with front paws in 3 weeks old *Ada*+/+ (n = 23), *Ada*−/− (n = 21) mice and *Ada*−/− mice treated with ERT for two weeks (n = 8); ANOVA **p < 0.005.

ADA enzymatic activity was not detected in the brain of ERT-treated Ada−/− mice, suggesting inability of PEG-ADA to cross the blood-brain-barrier (Fig. 6a). This fits reports that most PEGylated proteins do not leave the circulation or enter the brain^28^. Adenosine metabolite levels in the brain of PEG-ADA-treated mice were 10-fold lower than in untreated mice, but remained 3-fold higher than in wild-type (Fig. 6b). Peripheral detoxification by PEG-ADA can therefore passively lower adenosine levels in the brain, likely because metabolites exit from the brain by diffusion. ERT was able to fully correct ventriculomegaly observed in untreated Ada−/− mice (Fig. 6c–e). Similarly to Ada−/− mice, no other gross alterations were observed by brain histology in ERT-treated mice in comparison to wild-type (data not shown).

ERT-treated Ada−/− mice were also tested in the open field and dark/light test at PND15. PEG-ADA-treated Ada−/− mice showed a similarly decreased activity in the number of visits in the three zones as their untreated Ada−/− littermates (Fig. 6f). The observed hypoactivity correlated with a significantly reduced locomotor activity as shown by the total path covered (Fig. 6g). Moreover, the latency to go and spent time in the dark compartment was not improved in ERT-treated mice (Fig. 6h). No significant differences were found for both tests between untreated and treated Ada−/− mice, suggesting that ERT is insufficient to correct the observed abnormalities in exploration and anxiety-like behavior.

### Defects in adenosine receptor signaling contribute to observed alterations in Ada-deficient mice

Tonic activation of adenosine receptors has anxiolytic activity, while antagonists such as caffeine can cause anxiety^19^. Knockout of the Adora1 receptor in mice results in hyperalgesia and anxiety, including decreased exploration in the open-field and less time spent in the light portion of the Dark and Light Box^19^,^29^. Adora2a−/− mice display reduced exploratory activity, heightened anxiety, hypoalgesia, and aberrant locomotor responses to caffeine^30^, including an absent anxiogenic response to acute or chronic high-dose caffeine^19^,^31^.

In order to evaluate a possible contribution of Adora signaling to the observed anxiety-like behavior, we treated Ada+/+ and Ada−/− mice with 50 mg/kg of caffeine/day and tested their activity in the dark and light Box at PND15 (Fig. 6h). While Ada+/+ mice showed the expected increase in anxiety-like behavior, Ada−/− were indistinguishable from wild-type. The differences in the dark and light test observed between untreated Ada+/+ and Ada−/− mice therefore suggest defects in Adora signaling.

Adora1−/− animals exhibit hyperalgesia, whereas mice lacking Adora2a are hypoalgesic, indicating that adenosine may exert different effects on pain^32^. To better identify which Adora is involved in the Ada−/− phenotype, we performed Hot Plate tests in Ada−/− and wild-type mice to measure their pain perception. Ada−/− mice licked their hind paws significantly later than Ada+/+, which is a strong sign of discomfort in the hot-plate test (Fig. 6i). Ada−/− therefore show a similar phenotype to Adora2a−/− mice with reduced pain perception, suggesting defects in Adora2a signaling. Pain sensitivity showed a tendency to decrease after PEG-ADA treatment, but did not reach wild-type levels.

### Alterations in distribution and expression of Adora1 and Adora2a

**Figure 7:**
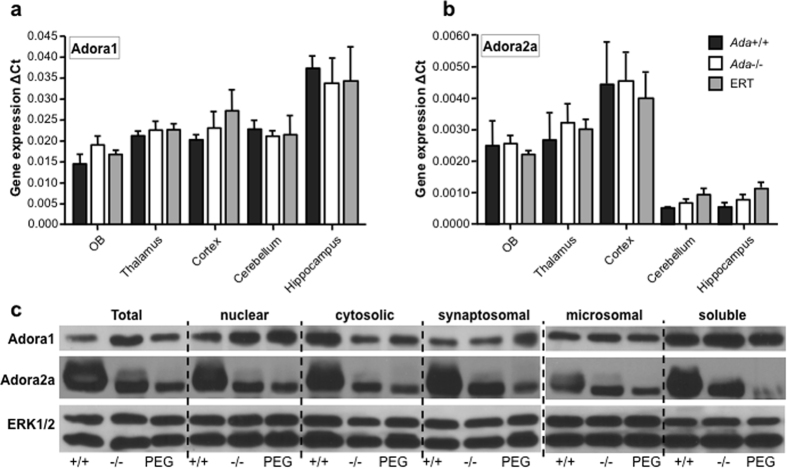
Alterations in adenosine A1 and A2a receptor expression in *Ada*−/− mice. (**a**) TaqMan gene expression analyses for adenosine receptor A1. Olfactory bulbs, thalamus, cortex, cerebellum and hippocampus *ex vivo*; ΔCt normalized for GADPH endogenous control (average of 6 experiments + SEM). (**b**) TaqMan gene expression analyses for adenosine receptor A2a. Olfactory bulbs, thalamus, cortex, cerebellum and hippocampus *ex vivo*; ΔCt normalized for GADPH endogenous control (average of 6 experiments + SEM). (**c**) Representative cropped Western Blot analysis for A1 (36 kDa), A2a (45 kDa) and ERK1/2 (42/44 kDa) as housekeeping control on brain fractions from a *Ada*+/+, *Ada*−/− and *Ada*−/− mouse treated with ERT. Total protein preparation, cell debris and nuclei (P1) and postnuclear supernatant (S1), synaptosomal fraction (P2) and supernatant (S2), microsomal pellet (P3) and post-microsomal soluble protein fraction (S3).

Since adenosine levels accumulate in Ada−/− mice, we assessed whether increased agonist exposure alters Adora expression levels in the brain. RNA expression levels of the Adora1 and Adora2a were not significantly different between wild-type, Ada−/− and PEG-ADA-treated mice (Fig. 7a,b). Almost no RNA expression was detected for Adora2b and Adora3 (data not shown).

The main regulatory pathway of adenosine receptors is post-transcriptional and involves phosphorylation of activated receptors by G protein-coupled receptor kinases. Upon agonist treatment, adenosine receptor subtypes are differently regulated. Adora1 are phosphorylated and internalized slowly, showing a typical half-life of several hours, whereas the Adora2a undergoes much faster downregulation, usually shorter than 1h^33^. We therefore addressed whether prolonged high adenosine exposure could lead to increased degradation of the Adora1 and Adora2a receptors. We separated membrane fractions from total brain of *Ada*+/+, *Ada*−/− and PEG-ADA treated mice and revealed the presence of Adora1 and Adora2a by Western Blot. Adora1 was present at comparable levels in Ada+/+, Ada−/− and treated membrane fractions, whereas Adora2a were decreased in Ada−/− as compared to Ada+/+ brains. Adora2a protein levels in PEG-ADA-treated mice remained low (Fig. 6c). Thereby suggesting aberrant Adora2a signaling in Ada−/− mice and that metabolic detoxification by ERT in the brain is insufficient to recover Adora2a degradation.

## Discussion

ADA-deficient patients who survive after allogeneic HSCT and HSC-GT display variable cognitive and behavioral alterations, but their pathogenesis had remained elusive^10^,^11^,^12^,^13^,^34^. Since Adoras are ubiquitously distributed in the CNS and adenosine acts as neuromodulator^16^ we hypothesized that adenosine, deoxyadenosine or their derivates mediate the neurological defects described in ADA-deficiency. By assessing neurological and behavioral features in Ada−/− mice and ADA-SCID patients, we obtained new insights into the role of ADA in brain function and the metabolic and molecular effects of adenosine accumulation. We report significant neurological and cognitive alterations in untreated as well as ERT-treated patients suggesting that these form an important part of the disease phenotype.

Neurological problems in ADA-SCID patients are rarely reported. Detoxification mediated by the materno-fetal circulation in utero may partially circumvent the metabolic deficit of the fetus, so that the neurological abnormalities manifest within a variable period after birth. Herein untreated ADA-SCID patients displayed several MRI abnormalities including enlargement of ventricles and subarachnoid spaces, WM alterations, motor dysfunctions and other instrumental alterations.

In Ada-deficient mice we describe unique neurological and behavioral characteristics correlating with the ADA metabolism and Adora signaling. Since the placenta of these mice was engineered to express Ada in uterus to rescue Ada−/− mice from prenatal lethality^22^, it is possible that the early alterations are less pronounced than in humans.

The effects of Ada deficiency in mice became more prominent in the later postnatal period, when ADA substrates accumulate^35^. We found that Ada-deficient mice were significantly less active and spent more time in the dark compartment. Despite their ability to move and pass the Rotarod test, they showed alterations in exploration and anxiety-like behavior.

Our evaluation in patients was conducted retrospectively, so no direct comparison between untreated and ERT-treated patients can be made. However we found that ADA-SCID patients on ERT have a high probability to manifest neurological (62%) and psychological (67%) alterations of different severity and independently from their past clinical history. Contrarily to previous reports we did not find any correlation between the severity of these manifestations with consanguinity of the parents or dAXP levels at diagnosis.

A low mental development index and IQ was measured in young as well as older ADA-SCID patients, indicating severe cognitive impairment, which is insufficiently controlled by PEG-ADA. Brain MR alterations in ADA-SCID patients included leukoencephalopathy and enlargement of ventricular and subarachnoid spaces^36^. Both were less frequent in older PEG-ADA-treated patients, suggesting that these manifestations are due to metabolic disruption and can be counteracted by extracellular metabolic detoxification. WM alterations on the other hand persisted in long-term treated patients, suggesting that these are cell-intrinsic defects. This is consistent with ADA-SCID patients after HSCT, which also continue to show MRI alterations^15^.

Comparative studies in the Ada-deficient mouse model allowed us to dissect the extrinsic metabolic from intrinsic cellular defects caused by the adenosine metabolism. The latter are not expected to be corrected by ERT. Ventriculomegaly in Ada−/− mice was improved by PEG-ADA treatment suggesting a metabolic adenosine-dependent nature of these alterations. Whereas the anxiety-like behavior and hypoactivity of Ada−/− mice were not improved by ERT, therefore indicating a cell-intrinsic or molecular defect. Caffeine exposure abolished the observed differences in anxiety-like behavior in Ada−/− compared to Ada+/+ mice, which suggested an involvement of Adora signaling in the Ada−/− behavioral phenotype.

Both in mice and humans, endogenous adenosine is a widely distributed upstream regulator of a broad spectrum of neurotransmitters, receptors, and signaling pathways^37^. Ada−/− shared several features with Adora1−/− and 2a−/− mice, such as ventriculomegaly, reduced exploratory activity, heightened anxiety^30^,^32^. However, only Ada−/− and Adora 2a−/− showed an absent anxiogenic response to acute or chronic high-dose caffeine^19^,^31^. Moreover, Ada−/− mice were less sensitive to pain, similarly to Adora2a-deficient mice.

Western Blot analysis for Adora2a protein expression confirmed these differences and showed no correction after PEG-ADA. We therefore concluded, that in Ada−/− mice Adora2a signaling rather than Adora1 signaling is impaired. It is conceivable, that the different post-transcriptional regulation of adenosine receptor subtypes upon agonist binding, i.e. slower for Adora1 and faster desensitization for Adora2a^33^, cause a stronger signaling impairment in the latter.

The reasons why adenosine receptor protein levels were not corrected by PEG-ADA remain to be elucidated. Evidence can be found in literature suggesting important cell surface interactions between ADA and Adoras^38^. The proposed physiological role of such protein–protein interactions is to make receptors more sensitive to adenosine. ADA binding was shown to allosterically affect the quaternary structure of Adora2a and to increase both agonist and antagonist binding^39^.

Although we did not observe gross differences in Adora1 expression in *Ada*−/− mice, a role for Adora1 involvement in the described neurological and behavioral phenotype cannot be excluded and may require further studies. In fact, human ADA was suggested to enhance the agonist and antagonist affinity also of the Adora1 receptor^40^. Adora2a and Adora1 oligomerize when co-expressed, suggesting that these two receptors might form part of a shared molecular transduction complex or signalosome^38^. Similarly, an additional role for other accumulating ADA metabolites cannot be excluded. There is a rapidly growing literature about the involvement of purinergic signaling and ATP in most disorders of the CNS^17^,^18^.

Overall, the persisting neurological defects described herein in PEG-ADA-treated patients and mice suggest that ADA delivery into the brain is required. The allosteric function of ADA in support of Adora signaling is unlikely to be supported by PEG-ADA, which does not cross the blood-brain barrier. Residual elevated adenosine levels might therefore continue to induce Adora2a degradation, since the receptor is not stabilized by allosteric ADA-binding^1^.

The persisting neurological and behavioral problems in patients after HSCT and HSC-GT suggest that even in the presence of systemic detoxification, blood derived ADA-expressing cells that cross the blood brain barrier do not deliver sufficient levels of ADA for full correction of the metabolic alterations in the brain^10^,^11^,^13^,^34^. Neurological alterations have been observed in patients receiving HSC-GT^12^,^41^, but further studies are needed to assess the extent of the metabolic corrections by endogenous ADA expression and subsequent improvement of these alterations. While treatment of ADA-SCID by HSCT or HSC-GT provides metabolic detoxification in the brain, it might be insufficient to provide the stabilizing allosteric support of ADA to Adora signaling locally. Direct delivery of ADA to the CNS through *in vivo* gene therapy might be explored as recently proposed for other metabolic disorders^42^,^43^.

Further studies will have to be performed to assess if a genotype-phenotype correlation exists between the ADA mutation and severity of the neurological phenotype, e.g. a mutation, which causes ADA protein expression without enzymatic activity may still support allosteric function in support of Adora signaling. Also certain Adora polymorphisms may lead to a different neurological outcome or may serve as prognostic markers to predict long-term outcome. Caffeine and related xanthines by binding to adenosine recognition sites can have significant behavioral effects on locomotor activity, learning and memory. In ADA-SCID patients carrying highly sensitive adenosine receptor subtypes or with poor outcome after treatment, adenosine receptor antagonists already available in clinical practice might be suitable to ameliorate their phenotype.

## Methods

### ADA-SCID patients and clinical trial

This is a retrospective study conducted on ADA-SCID patients referred to San Raffaele Hospital. They were evaluated for their neurological disease status by clinical and instrumental evaluations (see supplementary methods). The groups were composed of patients from different nationalities, cultures and clinical histories.

Patients or patients’ parents signed informed consent on anonymized data collection for research studies conducted at San Raffaele Hospital approved by the San Raffaele Scientific Institute’s Ethical Committee and Italian National Regulatory Authorities. For patients who subsequently underwent HSC-GT, part of the neurological analyses was performed as baseline evaluation. ADA-SCID gene therapy clinical trials were initially sponsored by the Telethon foundation (www.clinicaltrials.gov; #NCT00598481/#NCT00599781); in 2010 GSK has acquired the license for ADA-SCID gene therapy.

### Ada+/+ and Ada−/− mice

Ada-deficient mice have been described by Blackburn *et al*.^22^. Breeding pairs for FVB;129-Adatm1MW-TgN(PLADA)4118Rkmb were purchased from Jackson Laboratory (Bar Harbor, USA). Double-mutant mice for Adatm1MW or Ada+/+ mice were generated by intercrossing Ada+/− littermates. The genotype of the progeny was identified by PCR (www.jaxmice.jax.org). All animals were bred and maintained in a specific pathogen-free animal facility. All procedures were performed according to protocols approved by the Committee for Animal Care and Use of San Raffaele Scientific Institute (IACUC 490).

### Enzyme replacement therapy with PEG-ADA in Ada−/− mice

Ada−/− mice were injected intraperitoneally (i.p.) with PEG-ADA (Adagen, Enzon Inc., Piscataway, USA) at 1000 U (1 Unit is defined as the amount of enzyme necessary to convert 1 μM of adenosine to inosine per min at 25 °C) per kg body weight. Injections were started on PND10 and were given once a week.

### Caffeine treatment of Ada+/+ and Ada−/− mice

Ada+/+ and Ada−/− mice were injected intraperitoneally with caffeine (SIGMA) at 50 mg/kg/day. Injections were started on PND7 and were given once a day.

### Histological analyses

Hematoxylin and eosin stainings were performed on brains from 3-weeks-old intracardially perfused Ada+/+, Ada−/− or ERT-treated Ada−/− mice (see supplementary methods).

### Measurement of brain ADA activity and adenosine levels

Intracellular ADA enzymatic activity and adenosine levels were analyzed in total brain or brain parts by adenosine to inosine conversion followed by high-performance capillary electrophoresis (HPCE)^44^.

### FOX battery

Littermates were studied every 3 days until 18 days of age (postnatal days (PND) 3, 6, 9, 12, 15, 18). Each mouse was scored for the righting, crossed extensor and grasp reflexes, postural flexion, cliff drop aversion and negative geotaxis^27^.

### Functional and behavioral tests in mice

Ada+/+ and Ada−/− littermate mice were tested at PND15 and at PND20 for motor coordination on the rotarod, at PND20 for olfactory discrimination, at PND15 and PND20 for exploratory behavior in the open field, at PND15 for anxiety-like behavior in the dark/light test and at PND20 for pain reflexes in the hot plate test (see supplementary methods).

### Taqman Gene Expression

RNA was extracted and Real Time PCR reactions for Adora1, Adora2a, Adora2b and Adora3 were carried out as described^45^ (see supplementary methods).

### Western blot analysis

Brain samples from Ada+/+ and Ada−/− mice were prepared by standard methods or gradient centrifugation (see supplementary methods), subjected to sodium dodecyl sulfate-polyacrylamide gel electrophoresis (SDS-PAGE) and transferred to nitrocellulose membrane for labeling with anti-ADA, anti-Adora1, anti-Adora2a, anti-β-actin and anti-ERK1/2 antibodies (see supplementary methods).

## Additional Information

**How to cite this article:** Sauer, A. V. *et al*. Alterations in the brain adenosine metabolism cause behavioral and neurological impairment in ADA-deficient mice and patients. *Sci. Rep.* **7**, 40136; doi: 10.1038/srep40136 (2017).

**Publisher's note:** Springer Nature remains neutral with regard to jurisdictional claims in published maps and institutional affiliations.

## Supplementary Material

Supplementary Information

## Figures and Tables

**Table 1:** Summary of neurological and cognitive alterations detected in untreated ADA-SCID patients (n = 5) and patients under enzyme replacement therapy (n = 15).

	Sex	Neurological alterations	Behavioural and cognitive alterations	dAXP	Consanguineous parents	Age at last evaluation	Years of PEG-ADA at evaluation
*Untreated patients*							
Patient 1	F	MRI SS; EEG (1)		277	Yes	2.3	—
Patient 2	M	MRI SS	Eating disorder	450	No	1.0	—
Patient 3	F	MRI SS		138	Yes	1.5	—
Patient 4	M	MRI WM + SS; EEG (2); Motor delay (2), hypotonia (1), hyporeflexia (1), VEP latency (1)	Psychomotor retardation, verbal delay; eating disorder and logotherapy	6*	Yes	1.1	—
Patient 5	M	MRI WM		1639	Yes	0.3	—
				mean years:		1.2	
*ADA-SCID patients (<3yrs of age) treated with ERT*							
Patient 6	F		Hyperactivity and attention deficit	2	Yes	1.4	1.1
Patient 7	M	MRI WM + SS; Coordination deficit (2)	Verbal delay; hyperactivity and attention deficit; eating disorder and PEG from 36th MAB	5	No	1.3	0.8
Patient 8	F	MRI WM; Sensorineural hypoacusia (1)	Psychomotor retardation, verbal delay	23	Yes	2.8	2.7
Patient 9	M	Motor delay (1)		0	No	1.2	0.8
Patient 10	M	EEG (1)		0	Yes	1.2	0.8
Patient 11	F	VEP latency (1)		0	No	1.8	0.4
Patient 12	M	EEG (1); MRI WM + VV	Hyperactivity	74	No	1.9	1.3
Patient 13	M	MRI SS; EEG (1)		0	Yes	0.5	0.6
				mean years:		1.5	1.1
*ADA-SCID patients (>3yrs of age) treated with ERT*							
Patient 14	M	Sensorineural hypoacusia (1)	Verbal deficit; hyperactivity and attention deficit	5	Yes	5.3	5.3
Patient 15	M	Sensorineural hypoacusia, requiring hearing device (2)	Verbal deficit	0	No	5.1	5.0
Patient 16	M	Motor delay (1); EEG (1), epilepsy, under anti-epileptic treatment; MRI WM + VV; Sensorineural hypoacusia, requiring hearing device (2)	Verbal deficit	0	No	27.0	25.5
Patient 17	F	Motor delay (1); EEG (1)	Verbal deficit	3	No	16.0	15.9
Patient 18	F		Verbal deficit	0	No	25.0	23.2
Patient 19	M		Anxiety; Verbal deficit; hyperactivity and attention deficit; eating disorder	0	Yes	15.0	14.8
Patient 20	M	Motor delay (2); EEG (2), epilepsy, under anti-epileptic treatment	Verbal deficit	3	No	3.7	3.4
Patient 21	M		Verbal deficit; hyperactivity and attention deficit	0	Yes	8.0	8.0
				mean years:		13.1	12.6

(1) mild manifestations, (2) severe manifestations, dAXP = deoxyadenosine nucleotide measured in peripheral blood samples, N.D. = Not Done; BAER = brainstem auditory evoked responses, EEG = electroencephalography, ERT = enzyme replacement therapy, MRI = magnetic resonance imaging, PEG = percutaneous endoscopic gastrostomy, MAB = months after birth, SS = subarachnoid spaces, VEP = visual evoked potential, VV = ventricles, WM = white matter; *patient received transfusion of RBC at time of evaluation.
