# Supplementary material for: Alterations in the brain adenosine metabolism cause behavioral and neurological impairment in ADA-deficient mice and patients
Source: Sci Rep. 2017 Jan 11;7:40136. doi: 10.1038/srep40136 (PMC5225479; doi:10.1038/srep40136)
Supplement: Supplementary Information [file srep40136-s1.pdf]

SUPPLEMENTARY DATA: Alterations in the brain adenosine metabolism cause behavioral and neurological impairment in ADA-deficient mice and patients

Aisha V. Sauer, Raisa Jofra Hernandez, Francesca Fumagalli, Veronica Bianchi, Pietro L. Poliani, Chiara Dallatomasina, Elisa Riboni, Letterio S. Politi, Antonella Tabucchi, Filippo Carlucci, Miriam Casiraghi, Nicola Carriglio, Manuela Cominelli, Carlo Alberto Forcellini, Federica Barzaghi, Francesca Ferrua, Fabio Minicucci, Stefania Medaglini, Letizia Leocani, Giancarlo la Marca, Lucia D. Notarangelo, Chiara Azzari, Giancarlo Comi, Cristina Baldoli, Sabrina Canale, Maria Sessa, Patrizia D'Adamo and Alessandro Aiuti

Psychometric evaluation in ADA-SCID patients

In addition to the quantitatively assessed psychometric scores, we observed a variety of less quantifiable behavioral aspects during follow-up in ADA-SCID patients. Every child shows a different mode and intensity of these behavioral characteristics. Nevertheless, they represent noteworthy observations, which might prove useful for future psychological assessments of ADA-deficient individuals.

One of the aspects that most characterizes the patients in all three groups is their difficulty to behave according to their chronological age. This is particularly apparent in test settings requiring a minimum of internalized social norms and recognition of the figure and role of the adult. Most preschool children between 2 and 5 years of age, have difficulties to maintain attention for a sufficient amount of time during evaluation, manifesting motor restlessness and sometimes hyperkinesia. Patients struggle with the alternating test requirements and are unresponsive to corrections and admonitions both by the examiner and parents. They manifest little care of objects and difficulties to recognize the sense of ownership, thereby tending to take possession of test objects. Since these children are often hospitalized for a long time, most of them did not have the opportunity to attend kindergarten or their peers to learn how to adapt their own needs to those of others. It is very difficult to assess whether these aspects of hyperactivity and attention deficit are inherently correlated to the underlying disease or environmental limitations, since the disease manifests in an age in which learning takes place mainly by exposure and imitation of both adult and peers.

Another noteworthy behavioral aspect or better stage of development is weaning, in terms of autonomy and variety of food uptake. From anamnesis and conversations as

part of every psychological evaluation, we observed that in most of the children the passage to solid food intake has not been completed within the timing of weaning and has been met with resistance and difficulties. Many caregivers had to extend the period of weaning through prolonged periods of intermediate intake of semisolids without easily passing to permanently solid food intake. For some children transient parenteral nutrition was necessary. Also here, at least from a psychological point of view, it is difficult to assess whether the disease itself causes these difficulties in alimentation or whether the lack of learning during the oral phase of development can lead to complications in this regard. Due to the constant risk of infection in public places it is necessary that these children are raised without this important phase rich in stimuli through the oral exploration of objects around them. In fact, this learning phase is fundamental for the subsequent stage skills such as chewing food and swallowing. Every child shows a different mode and intensity of these behavioral characteristics. Nevertheless, they represent noteworthy observations, which might prove useful for future psychological assessments of ADA-deficient individuals.

SUPPLEMENTARY METHODS: Alterations in the brain adenosine metabolism cause behavioral and neurological impairment in ADA-deficient mice and patients

ADA-SCID patients and clinical trial

This is a retrospective study conducted on ADA-SCID patients referred to San Raffaele Hospital. They were evaluated for their neurological disease status by the below clinical and instrumental evaluations. The groups were composed of patients from different nationalities, cultures and clinical histories.

Patients or patients' parents signed informed consent on anonymized data collection for research studies conducted at San Raffaele Hospital approved by the San Raffaele Scientific Institute's Ethical Committee and Italian National Regulatory Authorities. All experiments were performed in accordance with relevant guidelines and regulations. For patients who subsequently underwent HSC-GT, part of the neurological analyses was performed as baseline evaluation. ADA-SCID gene therapy clinical trials were initially sponsored by the Telethon foundation (www.clinicaltrials.gov; #NCT00598481/#NCT00599781); in 2010 GSK has acquired the license for ADA-SCID gene therapy.

Patients were subjected to the following clinical evaluations:

- Standard neurological evaluation
- Psychometric evaluation (mental development index; total, verbal and performance intelligence quotient)
- Other neuropsychological variables (verbal deficits, hyperactivity and attention deficits, non autonomous alimentation)

We generally considered the test results at first evaluation for this study, with the exception of four long-term PEG-ADA treated patients, who's IQ was monitored over time.

During neurological examinations all patients were assessed for motor dysfunctions, such as coordination or deambulation deficits, alterations of muscle tone and trophism. ADA-SCID patients with one of the above manifestations were scored as suffering from mild motor dysfunctions, whereas more than one manifestations as severe motor dysfunctions (Figure 1).

Neuropsychological evaluations were performed during programmed visits to the Day Hospital in a silent structured room. A professional interpreter was involved to translate

the oral instructions given by examiners to Non-Italian native speakers. Depending on the patient's age, we used different scales for the psychometric assessments. Neuropsychological development, for children under 42 months, was assessed with the Bayley Scales of Infant Development-II edition (BSID-II), to obtain the Mental Developmental Index (MDI) as a measure of cognition. To obtain IQ measurements we used Wechsler Preschool and Primary Scale Of Intelligence (WPPSI) for children between 4 and 6 years of age; Wechsler Intelligence Scale for Children- Revised (WISC-R) or Wechsler Intelligence Scale for Children- third edition (WISC-III) for children between 6 and 16 years of age and Wechsler Adult Intelligence Scale- Revised (WAIS-R) for young adults over 16 years. The IQ and MDI are both expressed with a population average of 100 (SD ±15). For both scales 3 categories based on the average and standard deviation (SD) for each grade were defined: normal (-1 to +1 SD), borderline (-1 to -2 SD) and pathological (< -2 SD). Neuropsychological development, for children under 3 years, was assessed through general behavioral observations and application of the Bayley Scale of Infant Development (BSID-II). Upon BSID-II administration a raw score was obtained. Each patient's score was converted into a mental development index (MDI), normalized for their chronological age.

Some qualitative findings, which appeared noteworthy in our cohort of children with ADA SCID, were also considered in this study. Information on verbal deficits, hyperactivity and attention deficits were collected during psychometric assessments, when verbal and behavioral difficulties were observed. Information on non-autonomous alimentation was collected from the case history of each patient and is referred to difficulties in breast nutrition, in transition from liquid to solid foods or in regular alimentation in older children. Secondary observations due to medical complications were not considered for this study.

The following instrumental evaluations were performed in all three groups of patients: untreated, young and older PEG-ADA treated patients.

- BEAR
- EEG
- MRI
- VEP

However, as indicated in each panel of Figure 1, in some cases not the whole set of neurological and instrumental analyses was available.

Brainstem Auditory Evoked Responses (BAER)

Monaural clicks were delivered by a shielded earphone at 85 dBnHL, rate 21 Hz, stimulus duration 100 μsec; 2000 sweeps of 10 msec duration were averaged for each ear. Recording electrodes were placed at the ipsilateral and contralateral earlobes and referred to Cz (Fz was used as ground). The following parameters have been evaluated: absolute and interpeak latencies of waves I, III and V. Data have been compared with normative data from our laboratory.

EEG

Standard EEGs, with a duration of about 20 min, were acquired in resting awake condition, under control of vigilance, on a computer based system (Micromed System PLUS) from 19 standard 10/20 electrode locations (Fp1, F3, C3, P3, O1, F7, T3, T5, Fp2, F4, C4, P4, O2, F8, T4, T6) with linked ear reference (Homan et al., 1987); ground electrode was placed in Fpz. Data, filtered between 0.53 and 70 Hz, were digitized at 256 Hz and coded on 12 bits. Electrode-skin impedance was set below 5 kOhm for each electrode.

Brain MRI in ADA-SCID patients

MRI examinations were done on a 1.5T MR scanner. Standard sequences were performed with TURBO Spin Echo, Spin Echo, FLAIR and DWI techniques including: T2 and T1 weighted images with multiplanar sections, 4 and 3mm thick.

Flash Visual Evoked Potentials (VEP)

Flash stimulation with white light was performed using a light diffuser. White flashes were administered at 1 Hz; recording electrodes were placed using EEG conductive paste on Oz with reference electrode at Cz (ground electrode was placed at Fz). Latency of the IV component was measured and data was compared with normative data from our laboratory.

In vivo MRI in Ada-deficient mice

Brain imaging was performed at 3 weeks of age on a 7T-MRI scanner (Pharmascan, Bruker, Ettlingen, Germany). High-resolution coronal sections were acquired along the brain of mice maintained under anesthesia with flurane gas mixed with O2. A multislice

multiecho (MSME) sequence (TR_3465 ms and TE_18 and 44 ms) with a slice thickness of 0.85 mm was used to generate 15 contiguous T2-weighted images with a plane spatial resolution of 86_93_m2. For neuroanatomical analysis, the area of the measured regions was manually traced using Image J (National Institutes of Health, Bethesda, MD). The total volume of each structure was calculated by multiplying the sum of the measured areas by the slice thickness.

Histological analyses

Analyses were performed on brains from 3 weeks old intracardially perfused Ada+/+, Ada-/- or PEG-ADA treated Ada-/- mice. 2 µm thick sections from formalin-fixed, paraffin-embedded tissues were taken on a microtome and subjected to routine hematoxylin and eosin staining. Briefly, sections were de-waxed, re-hydrated and endogenous peroxidase activity blocked by 0.3% H_2O_2/methanol for 20 minutes. Heat-induced antigen retrieval was performed using a microwave-oven in 1 mM EDTA buffer (pH 8.0). Sections were then washed in Tris Buffered Saline (TBS) (pH 7.4) and incubated at room temperature for 1 hour in TBS/1% bovine serum albumin (BSA) with the specific primary antibody. Primary antibodies used were: mouse anti-NeuN (1:1500, Chemicon), rabbit anti-GFAP (1:200, Dako), mouse anti-CNPase (1:1000, Chemicon). Signal has been revealed using the DAKO Envision+System-HRP Labelled Polymer Anti-Rabbit or Anti-Mouse, followed by Diaminobenzydine (DAB) as chromogen and Hematoxylin as counterstain Images were acquired with an Olympus DP70 digital camera mounted on an Olympus Bx60 microscope, using CellF imaging software (Soft Imaging System GmbH).

FOX battery

Littermate mice were studied every 3 days until 18 days of age (postnatal days 3, 6, 9, 12, 15, 18). Growth rates were measured at every test point. Each mouse was scored for the righting reflex consisting on placing the mouse on its side and waiting until it returns to the normal position with all four paws on the ground. For the crossed extensor reflex, one hind limb is pinched leading to its flexion and extension of the opposite hind limb. In the grasp reflex when a mouse's limb is struck with an object, the mouse grasps it. For the postural flexion, a mouse suspended by the tail opens the anterior limbs and turns its body on one side. In the cliff drop aversion test, a mouse is placed on the edge of a table with the forepaws and snout over the edge; the normal response is to turn and crawl

away from the cliff drop. The negative geotaxis scores the mouse's ability to turn around and crawl up from a 45° slope when it is placed with its head pointing down.

Rotarod test

Littermate mice were tested for motor coordination at PND15 and at PND20 in the rotarod test. The rotarod is an apparatus composed by a horizontal rotating rod (diameter of approximately 3cm) on which the mouse has to keep its balance. Five mice are simultaneously placed on the rotarod apparatus, separated by large disks. In the accelerating rotarod test each mouse was subjected to 5 consecutive trials, with an interval of 15min. Mice were placed every trial on the rotarod apparatus with the rod rotating at 4rpm (rotations/minute) during the first minute, then the rotation speed is increased every 30sec by 4 rpm reaching the maximum speed of 36rpm. A trial ended for a mouse when it fell down or when 200secs were completed.

Olfactory discrimination task

Littermate mice were tested at PND20 for the olfactory discrimination test. A modified home cage was built to allow the separation of used and new bedding. Two plexiglass inserts were slid into the center of a cage and held into place by a plexiglass track mounted on either side of the cage, thus separating the cage into three areas. One side of the modified home cage was filled with new bedding up to the level of the insert, and the other side was filled with the bedding from the home cage that had not been changed for at least 4 days prior to testing, and thus was soiled. The smaller central area in between the plexiglass inserts was used as 'neutral' starting point. During the 5min trial mice had unrestricted access to either side of the modified home cage and the percentage of time spent in each compartment was recorded. After 30min of resting, mice were retested for another 5min.

Open field test

Exploratory behavior of independent groups of littermate mice were tested at PND15 and PND20 in the open field test. Illumination in the room was by indirect diffuse room light (4x40W bulbs, 12lx). The mice were introduced into the empty arena and observed for 30 minutes. For time course analysis, the total observation time was portioned into three periods of 10min (t1-t3). For quantification of exploration activity, arena was divided three different zones: *exploration*, *home* and *intermediate* zone (Madani et al.,

2003). The exploration zone corresponds to the more aversive area of the arena, like the center zone in the open field. The home zone is the preferred part of the arena, corresponding to the wall zone. The remaining areas are defined as intermediate zone. Exploration activity such as the total path length and number of visits in different zones of the arena were scored.

Dark/light box

Littermate mice were tested at PND15 for anxiety-like behavior in the dark/light test. A 20×30cm lit chamber with transparent Perspex walls (20cm high) and open top was connected to a 20×15×20cm plastic dark box, which was completely closed except for the 7.5×7.5cm door connecting to the lit chamber. Illumination was by direct room light (500 lx). Each mouse was released in the middle of the lit compartment and observed for 5 min.

Hot plate test

Pain reflexes in response to a thermal stimulus were measured by the hot plate test in littermate mice at PND20. The surface of the hot plate is heated to a constant temperature of 52°C, as measured by a built-in digital thermometer with an accuracy of 0.1^{o}C and verified by a surface thermometer. Mice are placed on the hot plate (15cm diameter), which is surrounded by a clear acrylic cage (19cm tall, open top), and the Start/Stop button on the timer is activated. The latency to respond to either lick a paw or to rise on hind legs is measured to the nearest 0.1 seconds by deactivating the timer when the response is observed. The mouse is immediately removed from the hot plate and returned to its home cage. If a mouse does not respond within 60 seconds, the test is terminated and the mouse is removed from the hot plate. Animals are tested only once, one at a time and are not habituated to the apparatus prior to testing.

Video tracking, data collection and statistical analysis of functional and behavioral tests

For exploration tests (Open Field, Dark/Light box) animals were video-tracked using the EthoVision 2.3 system (Noldus Information Technology, Wageningen, the Netherlands, http://www.noldus.com) using an image frequency of 4.2/s. Raw data were transferred to Wintrack 2.4 (http://www.dpwolfer.ch/wintrack) for off-line analysis. Statistical

computations were done using Statview 5.0 (SAS Institute, Cary, NC, USA, www.statview.com).

Taqman Gene Expression

For all genes of interest specific Taqman primer-probe sets purchased as Assay-on-Demand for gene expression from Applied Biosystems (Foster City, CA). The relative expression amount of each gene of interest was normalized in respect to the expression of Glyceraldehyde 3-phosphate dehydrogenase (Gadph), used as an endogenous control. Genes of Interest (Assay on demand): Adora1 (Mm01308023_m1), Adora2a (Mm00802075_m1), Adora2b (Mm00839292_m1), Adora3 (Mm00802076_m1).

Sucrose and glycerol gradient centrifugation

Briefly, brains were homogenized in buffered sucrose (320mM sucrose, 2mM DTT, 1mM EGTA, 1mM EDTA, 4mM HEPES KOH, pH 7.4) supplemented with protease inhibitors by twelve strokes in a Teflon-glass homogenizer. Cell debris and nuclei (P1) were removed by centrifugation for 10 min at 1,100g. The postnuclear supernatant (S1) was centrifuged for 10 min at 9,200g, and the resulting pellet was resuspended in the buffered sucrose. The resuspended pellet was further centrifuged for 15 min at 10,200g to obtain the washed synaptosomal fraction (P2). Supernatant from the medium-speed centrifugation was centrifuged for 2 h at 167,000g to obtain microsomal pellet (P3) and soluble protein fraction (S3). The washed P2 was lysed by addition of 10 volumes of water and homogenization in a Teflon-glass homogenizer. The synaptosomal membrane-enriched fraction (LP1) was collected by centrifugation at 25,000g for 20 min. The supernatant (LS1) was further centrifuged at 165,000g for 2 h to sediment LP2 (synaptic vesicle-enriched fraction), leaving the remaining soluble fraction, LS2. Fractions were snap-frozen in liquid nitrogen and stored at −80°C until use.

Western blot analysis

Brain samples from Ada+/+ and Ada-/- mice prepared by standard methods or gradient centrifugation were subjected to sodium dodecyl sulfate-polyacrylamide gel electrophoresis (SDS-PAGE), and transferred to nitrocellulose membrane. Primary antibodies used were: rabbit anti-ADA (H-300) and rabbit anti-Adora2a (sc-32261) were obtained from Santa Cruz Biotechnology (Santa Cruz, CA); rabbit anti-Adora1 (ab82477) from Abcam (Cambridge, UK); anti-glyceraldehyde-3-phosphate dehydrogenase

(Gadph) from Chemicon (Temecula, CA) and anti-ERK1/2 (9101) from Cell Signaling Technology (Beverly, MA) were used as loading control. Horseradish peroxidase-conjugated secondary antibodies from DAKO (Glostrup, Denmark).

Untreated patients	**MRI**	**White matter abnormalities associated with ADA deficiency**	**Ventricles (VV) and subarachnoid space (SS)**	**Collateral findings**
Patient 1	normal		Slightly increased size of SS	Sinusitis
Patient 2	normal		Slightly increased size of SS	
Patient 3	normal		Slightly increased size of SS	Arnold-Chiari syndrome I
Patient 4	altered	Hypomyelination	Slightly increased size of SS	CC thinned, small pituitary gland, sinusitis
Patient 5	altered	Mild hypomyelination		CC slightly thinned, small pituitary gland
ADA-SCID patients (<3yrs of age) treated with PEG-ADA				
Patient 6	normal			Sinusitis
Patient 7	altered	Small white matter abnormalities in the periventricular areas and in the radiate crowns	Slightly increased size of SS	
Patient 8	altered	Signal alterations of cortico-pial membrane with dural thickening, mild hypomyelination		
Patient 9	normal			
Patient 10	normal			
Patient 11	normal			
Patient 12	altered	Diffuse white matter alterations	Enlarged lateral VV	
Patient 13	normal		Slightly increased size of SS	
ADA-SCID patients (>3yrs of age) treated with PEG-ADA				
Patient 14	normal			Mastoiditis left
Patient 15	normal			
Patient 16	altered	Periventricular alterations in white matter	Very enlarged and dysmorphic VV	
Patient 17	normal			Hydrops of optic nerves, empty sella, sinusitis
Patient 18	N.D.			
Patient 19	normal			
Patient 20	N.D.			
Patient 21	normal			

Supplementary Table 1: Brain MRI performed in untreated ADA-SCID patients (n=5) and patients under enzyme replacement therapy (n=15)
CC= corpus callosum, VV= ventricles, SS= subarachnoid spaces, N.D.= Not Done

			Ada-/-			
**PND**	**RR**	**CE**	**GR**	**PF**	**CD**	**NG**
3	0	1	0	0	N.D.	N.D.
6	0	0	0	0	1	N.D.
9	0	0	0	0	3	N.D.
12	0	0	0	0	1	2
15	0	0	0	0	0	0
18	0	1	0	0	0	2
21	0	3	0	0	0	3

Supplementary Table 2: FOX battery test performed in *Ada*+/+ (n=21) and *Ada*-/- (n=13) mice at postnatal day (PND) 3, 6, 9, 12, 15, 18 and 21
RR= Righting reflex, CE= Crossed extensor, GR= Grasp reflex, PF= Postural flexion, CD= Cliff drop aversion, NG= Negative geotaxis
Ada+/+ mice passed all tests successfully. Indicated is the number of *Ada*-/- mice that did not pass each test.
N.D.= Not performed at early age. At PND3 all mice had no whiskers. All mice opened their eyes at PND12.
